# Pre-exposure prophylaxis against HIV infection among male population under perceived risk of sexually transmitted infections in Vojvodina, Serbia: a cross-sectional study on knowledge and attitudes

**DOI:** 10.3389/frph.2026.1773596

**Published:** 2026-03-11

**Authors:** Vladimir Vuković, Svetlana Ilić, Mioljub Ristić, Farida Bassioni-Stamenić, Radmila Zobenica, Smiljana Rajčević, Ružica Cvetićanin, Olivera Stanišić, Tatjana Medić, Jasmina Jandrić Kočić, Nebojša Bohucki, Svetlana Popov, Danijela Simić, Zoran Milosavljević, Snežana Brkić, Daniela Marić

**Affiliations:** 1 Institute of Public Health of Vojvodina, Novi Sad, Serbia; 2 Faculty of Medicine, University of Novi Sad, Novi Sad, Serbia; 3 Faculty of Medicine, University of Belgrade, Belgrade, Serbia; 4 Public Health Institute Kikinda, Kikinda, Serbia; 5 Public Health Institute Pančevo, Pančevo, Serbia; 6 Public Health Institute Sombor, Sombor, Serbia; 7 Public Health Institute Sremska Mitrovica, Sremska Mitrovica, Serbia; 8 Public Health Institute Subotica, Subotica, Serbia; 9 Public Health Institute Zrenjanin, Zrenjanin, Serbia; 10 Institute of Public Health of Serbia “Dr Milan Jovanović Batut”, Belgrade, Serbia; 11 Clinic for Infectious Diseases, University Clinical Center of Vojvodina, Novi Sad, Serbia

**Keywords:** attitude, HIV, knowledge, men at risk, pre-exposure prophylaxis (PrEP), prevention

## Abstract

**Introduction:**

Globally, awareness and use of pre-exposure prophylaxis (PrEP) for preventing HIV vary widely. This cross-sectional study assessed knowledge, attitudes, and experiences related to PrEP use among sexually active men in Vojvodina, Serbia.

**Methods:**

Study was conducted from July to December 2024. Data were collected by seven district public health institutes in Vojvodina and two NGO community checkpoints. Participants were adult men with perceived high-risk sexual behaviour, infection symptoms, or seeking sexual health advice, regardless of their sexual orientation. Participation was anonymous and involved a structured self-administered questionnaire.

**Results:**

Among 597 participants (28.3% of clients counselled and/or tested across nine centres), 71.0% were aware of PrEP and 17.2% had previously used it. About 55% of participants had correct basic PrEP knowledge, 28.8% incomplete, and 18.8% insufficient. Overall, 80.2% expressed a positive attitude toward PrEP, 11.7% were undecided, and 8.1% have negative attitude. Age categories (31–40 years: OR = 1.66, 95% CI: 1.01–2.73, *p* = 0.047), consistent use of online apps to meet partners (OR = 1.98, 95% CI: 1.09–3.62, *p* = 0.026), and prior PrEP use (OR = 2.12, 95% CI:1.33–3.40, *p* = 0.002) were associated with higher odds of correct PrEP knowledge. Correct knowledge was the strongest predictor of a positive attitude toward PrEP (OR = 3.21, 95% CI: 1.68–6.12, *p* < 0.001).

**Discussion:**

Despite high awareness, use of PrEP was limited, and accurate knowledge strongly predicted positive attitudes, underscoring the importance of education and counselling. Enhancing public health capacity, reducing stigma, and improving information flow are essential for effective, equitable HIV prevention in Serbia and the wider Central and Eastern Europe region.

## 1 Introduction

Numerous studies have confirmed that pre-exposure prophylaxis (PrEP) is a highly effective biomedical intervention for reducing new HIV infections among populations at increased risk when used correctly and consistently (1–5). Its effectiveness has been demonstrated across diverse populations—including men who have sex with men (MSM), heterosexual individuals, and people who inject drugs—particularly in high-income settings where implementation and adherence support have been strong (5, 6). Clinical trials such as “IPERGAY” and “PROUD” showed that PrEP can reduce HIV acquisition by up to 86% among MSM when used either daily or on demand (4). Economic modelling further suggests that PrEP contributes to substantial health benefits and cost savings by reducing HIV incidence among individuals who would otherwise require lifelong antiretroviral therapy (5, 7, 8). Consequently, PrEP has become a cornerstone of combination prevention strategies, complementing HIV testing, counselling, and treatment-as-prevention approaches (5, 8).

Across Europe, the introduction of PrEP has been gradual, with increasing numbers of countries licensing it for preventive use since 2016 (4, 5, 8). However, uptake remains below the level required to significantly impact epidemic control, with estimates ranging from 0.5 to 3.6 users per 100,000 adults in many European Union member states, compared to up to 6.2 per 100,000 in the United States (5, 8, 9). While awareness and willingness to use PrEP have grown, there remains a pronounced “PrEP gap”—the discrepancy between those who would consider using it and those who actually do—which was estimated at 17.4% across EU countries in 2019, with the highest levels reported in Central and Eastern Europe (CEE) (4).

In CEE, lower reported HIV prevalence has delayed national prioritization of PrEP, despite substantial donor-supported HIV prevention efforts, highlighting a persistent gap between available resources and their integration into routine prevention services (4, 5, 8, 10). The persistent challenge lies in limited system-level integration, policy inconsistency, and sociocultural barriers that hinder sustainable and inclusive HIV prevention programs (8, 11). Stigma, discrimination, and moralizing public discourse surrounding sexuality and HIV continue to constrain access to prevention services, especially among MSM and other key populations (11, 12). These systemic and social determinants have slowed the normalization of PrEP as a public health tool across CEE compared to Western Europe, where national PrEP programs and public funding mechanisms are already well established (4, 5). In Serbia, 177 new HIV diagnoses were reported in 2023, corresponding to an incidence rate of 2.66 per 100,000 population, and the national surveillance data indicate an overall upward trend in HIV incidence over the past decade, with rates increasing from 1.93 in 2014 to a peak of 3.21 per 100,000 in 2019 (13). Surveillance data further indicate a pronounced male predominance, with a male-to-female ratio of 9:1 among newly diagnosed cases in 2023. Although PrEP has been formally included in the Serbian national clinical guidelines, it has not been fully integrated into routine, widely accessible public health services, nor supported through a comprehensive national PrEP program with stable public reimbursement (14). During the study period, access to PrEP remained limited and largely dependent on individual initiative, selected clinical settings, or NGO-mediated counselling and referral pathways rather than standardized nationwide provision.

Globally, substantial variation persists in PrEP awareness and uptake. Studies indicate that awareness among MSM typically ranges between 20% and 30%, although willingness to use PrEP often exceeds 80% once individuals are informed about its effectiveness (6, 15). For example, in a multicenter study conducted in China, only 22.7% of MSM had heard of PrEP, yet 89.5% expressed willingness to use it (15). In Italy, 87% of MSM were aware of PrEP, but only 7.5% had ever used it, with barriers including cost, low perceived risk, fear of side effects, and stigma (16). These discrepancies illustrate that awareness alone does not translate into use and highlight the need for structural, educational, and stigma-reduction interventions to bridge the awareness–uptake gap.

In Serbia, HIV incidence has remained relatively stable over the past decade, with newest infections occurring among MSM and other key populations at increased risk (12). Despite progress in expanding voluntary counselling and testing and improving treatment coverage, PrEP awareness and use remain limited. Community-based research and NGO data suggest that knowledge and positive attitudes toward PrEP are more common among younger, urban, and better-educated men, especially those connected to civil society organizations or online health information networks (6, 16). However, awareness and confidence among healthcare providers to recommend or prescribe PrEP remain low, as shown in both European and international studies (17, 18). These patterns underscore the need for education among healthcare workers and the public alike, as well as for the integration of PrEP within broader sexual health frameworks to ensure equitable access and uptake in Serbia and the wider CEE region.

However, data from the Balkan region remain very limited. To date, no large-scale studies have explored awareness and attitudes toward PrEP among sexually active men in Serbia. Therefore, within the framework of this study, the main goal was to assess the level of knowledge, attitudes, and previous experiences related to PrEP use among individuals with perceived high-risk sexual behaviour in the Autonomous Province of Vojvodina (AP Vojvodina, northern Serbia) during the period preceding the planned wide-spread availability of PrEP. In addition, we aimed to identify areas where targeted health education on the proper use of PrEP is most needed, in order to further raise the public's awareness of its importance and benefits.

## 2 Methods

### 2.1 Study participants and data collection

This cross-sectional study was conducted between July 3 and December 31, 2024, at the Institute of Public Health of Vojvodina (IPHV), Centre for Disease Control and Prevention, Department for Epidemiological Surveillance of HIV, Hepatitis, and sexually transmitted infections (STIs). The study was additionally implemented through a network of regional public health institutes across the Autonomous Province of Vojvodina (Kikinda, Pančevo, Sombor, Sremska Mitrovica, Subotica, and Zrenjanin), thus covering all seven administrative districts of the province, as well as at community-based checkpoints managed by the NGOs “Razvojni centar preventivnih međusektorskih usluga—PRIMUS” and “Association Red Line” (“Udruženje Crvena linija”).

The study population comprised adult male clients aged ≥18 years who reported perceived high-risk sexual behaviour, symptom/s of STIs, or sought sexual health check-ups, regardless of their sexual orientation. Participants were recruited using a non-probability sampling approach among individuals attending voluntary, confidential counselling and testing for STIs at one of the participating centres or community checkpoints in the AP Vojvodina, during the study period.

Participation involved completion of a structured, self-administered questionnaire assessing knowledge and attitudes toward the use of PrEP for HIV prevention. Prior to large-scale implementation, the questionnaire was piloted with 20 respondents at the IPHV to evaluate its clarity, comprehensiveness, and response option suitability.

The first section of the questionnaire included 11 items covering socio-demographic characteristics and indicators of the STI risk. Risk assessment was based on partnership status, condom use, use of psychoactive substances during sex (including chemsex practice), HIV testing frequency within the past year, and use of online applications to meet sexual partners. The second section evaluated PrEP knowledge using eight statements addressing core information about PrEP's mechanism of action, efficacy, and recommended use. Each statement offered three response options—affirmative (true), negative (false), and uncertain (I don't know)—allowing differentiation between general awareness of PrEP and evidence-based understanding of its preventive role. The third section assessed previous experience with PrEP use (if any), while the final section examined attitudes toward PrEP through five statements rated on a five-point Likert scale from 1 (“completely disagree”) to 5 (“completely agree”).

Exclusion criteria included females, participants under 18 years of age, lack of informed consent and completion of less than 50% of the questionnaire items. Finally, only those that responded affirmative to the first question “Are you completing this questionnaire for the first time?” were included in the study. Each participant was assigned a unique identification code, and responses were entered into a secure, password-protected electronic database accessible only to members of the research team.

### 2.2 Ethical considerations

The study protocol was approved by the Ethics Committee of the Institute of Public Health of Vojvodina (approval number 01-1002/2, July 3, 2024). Participation was voluntary, anonymous, and uncompensated. Written informed consent was obtained by selecting the “YES” box in the “Consent to participate in the study” section to the statement “I have read and fully understood the text about the purpose and procedure of this study on knowledge and attitudes regarding the use of PrEP, and I agree to participate in this short survey” at the beginning of the questionnaire.

### 2.3 Statistical analyses

Descriptive statistics were used to summarize the data, presented as means with standard deviations (SD) for continuous variables, and as frequencies and percentages for categorical variables. Participants were categorized into four age groups: 18–25, 26–30, 31–40, and >40 years.

The PrEP experience and PrEP consideration to use cascades were operationalized based on a previously published framework by Wirawan GBS et al. (19). The PrEP experience cascade consisted of PrEP awareness (“Have you heard of the PrEP before participating in this study?”) and lifetime PrEP use (“Have you ever used PrEP?”). Participants who reported PrEP awareness but no prior PrEP use (i.e., PrEP-naïve participants) were subsequently included in the PrEP consideration cascade. This cascade comprised two sequential steps: engagement in at least one form of risky sexual behaviour in the past year associated with an increased risk of HIV acquisition, and consideration of PrEP use as a preventive measure against HIV (“Have you ever considered using PrEP as a preventative measure against HIV?”).

To evaluate overall risk behaviour, participants were classified as being at increased risk of HIV acquisition if they reported at least one of the following behaviours during the past year: (1) sexual partnerships (having a partner they barely know or having a regular partner in an open relationship), (2) condom use (occasional or never), and (3) use of psychoactive substances during sex, including chemsex practice (sometimes or always).

Participants were also asked whether they had ever heard of PrEP. Those who responded positively were further categorized based on two knowledge items: (a) “When taken correctly, PrEP is about 99% effective in preventing HIV,” and (b) “PrEP can be taken daily or before and after sex according to a specific regimen.” Participants affirming both statements were considered to have correct basic knowledge; those affirming only one, incomplete basic knowledge; and those affirming neither, incorrect knowledge about PrEP. General attitude toward PrEP was assessed through the statement: “I believe that the use of PrEP is an important method in the prevention of HIV infection,” categorized as affirmative (agree or strongly agree), undecided (neither agree nor disagree), or negative (partially or completely disagree).

Kruskal–Wallis test was used for the comparison of continuous variables, whilе the differences in categorical variables were assessed using the Chi-square test or Fisher's exact test, as appropriate. Univariate and multivariate logistic regression analyses were used to examine potential predictors of having a better basic knowledge of the PrEP and having a positive attitude toward the PrEP. Variables included in the multivariable models were selected based on a combination of statistical significance in univariable analyses and theoretical considerations regarding their potential confounding effects, and ultimately, the models were adjusted for age and marital status. Statistical significance was set at *p* < 0.05 (two-tailed). All statistical analyses were performed using the statistical software STATA v.17.0 (College Station, TX, USA: StataCorp LLC 2021).

## 3 Results

**Figure 1:**
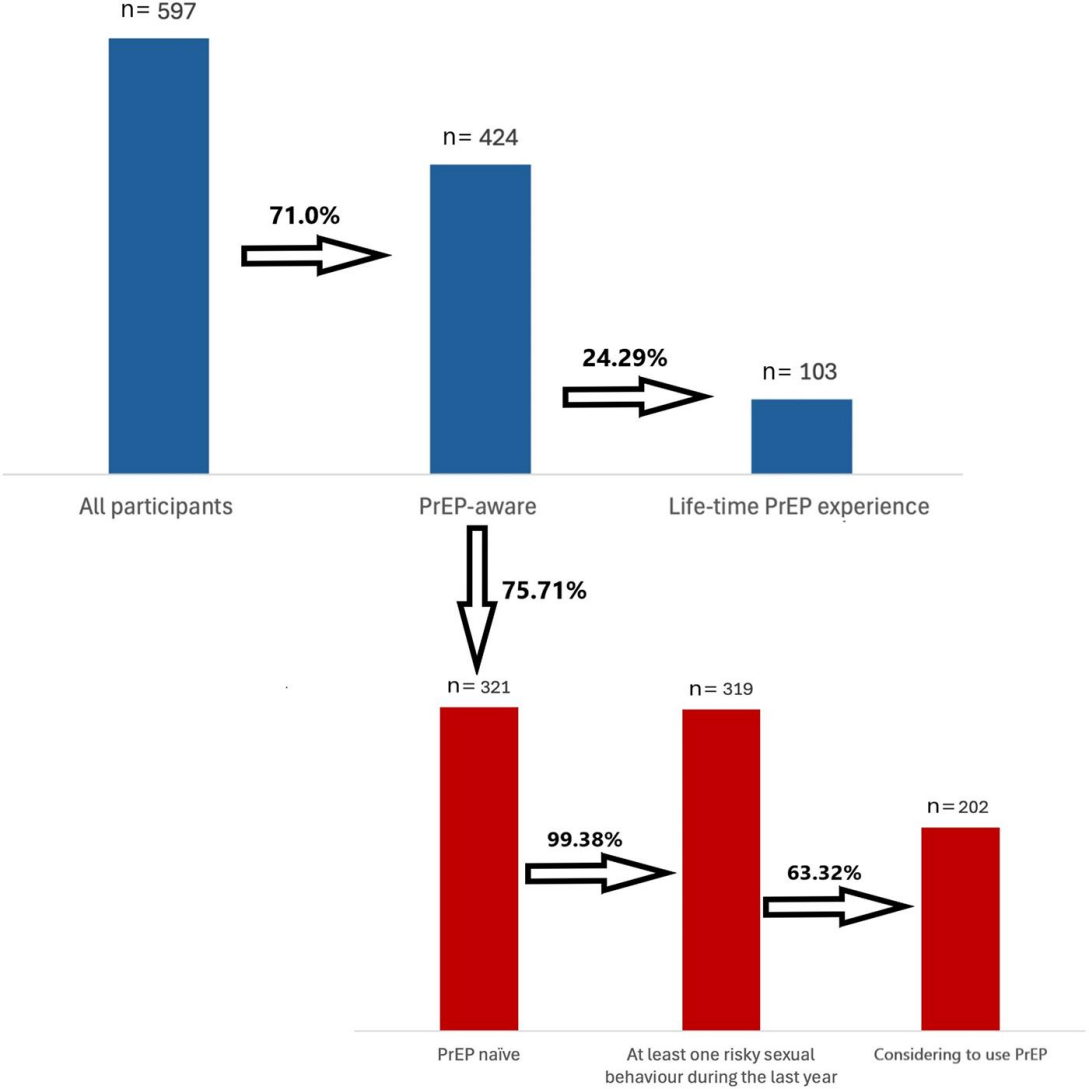
PrEP experience and consideration to use PrEP cascades. % indicates the proportion of participants who progressed to the subsequent stage within the cascade framework.

During the six-month study period, 597 participants were included, representing 28.3% of all clients counselled and/or tested across nine recruiting centres (*N* = 2111). The majority of participants were aware of PrEP (*n* = 424, 71%) as shown in Figure 1. Among those aware of PrEP, 103 individuals (24.29%) reported previous PrEP use. The remaining 321 (75.71%) PrEP-aware participants had never used PrEP (PrEP-naïve). Of these PrEP-naïve participants, 319 (99.38%) reported engagement in at least one form of risky sexual behaviour in the past year associated with an increased risk of HIV acquisition. Among PrEP-naïve participants who engaged in such risky behaviour, 202 (63.32%) reported having considered PrEP as a preventive measure against HIV infection.

The majority of participants were recruited through NGOs (77.6%), as presented in the Table 1. Mean age of the participants was 31.7 years (SD = 8.64); the largest age group was 31–40 years (31.8%), followed by 18–25 years (29.5%). Regarding education, most participants had completed high school (40.4%), followed by those with a university degree (34.5%). Most participants were unmarried (62.3%) and employed (68.3%). With respect to STI risk, 28.3% reported a permanent monogamous sexual partner and 26.3% a permanent partner with whom is in an open relationship. More than half reported occasional condom uses overall. Regarding the HIV testing, one in four participants (25.6%) had not been tested for HIV in the past year. More than a half (59.0%) reported occasional use of online apps to meet new partners, and nearly all participants (99.5%) reported at least one of the listed risky behaviours.

**Table 1:** General characteristics of the participants and the evaluation of their risk of contracting STIs.

		Have you heard of the PrEP before participating in this study? (*n* = 597)			*p*-value^a^
	Total	No (*n* = 173, 29.0%)	Yes, and used it (*n* = 103, 17.20%)	Yes, but did not use it (*n* = 321, 53.80%)	
Testing setting					
Public health institute	134 (22.45)	26 (15.03)	22 (21.36)	86 (26.79)	**0** **.** **011**
NGO	463 (77.55)	147 (84.97)	81 (78.64)	235 (73.21)	
Age in years, mean (SD)	31.7 (8.64)	32.54 (9.57)	32.60 (8.07)	30.96 (8.24)	0.099
Age category (years)					
18–25	176 (29.48)	55 (31.79)	21 (20.39)	100 (31.15)	**0** **.** **007**
26–30	127 (21.27)	21 (12.14)	27 (26.21)	79 (24.61)	
31–40	190 (31.83)	59 (34.1)	37 (35.92)	94 (29.28)	
>40	104 (17.42)	38 (21.97)	18 (17.48)	48 (14.95)	
The highest completed level of education					
Elementary school	19 (3.18)	9 (5.2)	1 (0.97)	9 (2.80)	**0** **.** **011**
High school	241 (40.37)	72 (41.62)	33 (32.04)	136 (42.37)	
College/Higher school	131 (21.94)	47 (27.17)	26 (25.24)	58 (18.07)	
Faculty	206 (34.51)	45 (26.01)	43 (41.75)	118 (36.76)	
Marital status					
Married	22 (3.69)	5 (2.89)	5 (4.85)	12 (3.74)	**<0** **.** **001**
Unmarried	372 (62.31)	86 (49.71)	58 (56.31)	228 (71.03)	
Common-law partnership	126 (21.11)	45 (26.01)	25 (24.27)	56 (17.45)	
Divorced	63 (10.55)	31 (17.92)	13 (12.62)	19 (5.92)	
Widower	10 (1.68)	4 (2.31)	2 (1.94)	4 (1.25)	
Missing	4 (0.67)	2 (1.16)	0	2 (0.62)	
Working status					
Pupil/student	76 (12.73)	19 (10.98)	9 (8.74)	48 (14.95)	0.311
Employed	408 (68.34)	115 (66.47)	77 (74.76)	216 (67.29)	
Unemployed	101 (16.92)	33 (19.08)	16 (15.53)	52 (16.2)	
Missing	12 (2.01)	6 (3.47)	1 (0.97)	5 (1.56)	
*Risk assessment for STIs during the last year*					
During this period, I have:					
Permanent sexual partner	169 (28.31)	43 (24.86)	31 (30.1)	95 (29.6)	**0** **.** **049**
Permanent sexual partner with whom I am in an open relationship	157 (26.3)	46 (26.59)	31 (30.1)	80 (24.92)	
Sexual partners whom I barely know	131 (21.94)	29 (16.76)	22 (21.36)	80 (24.92)	
I don't have a sexual partner	140 (23.45)	55 (31.79)	19 (18.45)	66 (20.56)	
I use a condom during sexual intercourse					
Always	216 (36.18)	53 (30.64)	36 (34.95)	127 (39.56)	**0** **.** **017**
Occasionally	301 (50.42)	94 (54.34)	45 (43.69)	162 (50.47)	
Never	80 (13.4)	26 (15.03)	22 (21.36)	32 (9.97)	
During sexual intercourse, I use psychoactive substances (including chemsex practice)					
Always	7 (1.17)	3 (1.73)	1 (0.97)	3 (0.93)	**0** **.** **003**
Occasionally	219 (36.68)	68 (39.31)	52 (50.49)	99 (30.84)	
Never	371 (62.14)	102 (58.96)	50 (48.54)	219 (68.22)	
In the last year, how often have you been tested for HIV?					
Never	153 (25.63)	60 (34.68)	14 (13.59)	79 (24.61)	**<0** **.** **001**
1–2 times	281 (47.07)	68 (39.31)	41 (39.81)	172 (53.58)	
3–5 times	141 (23.62)	39 (22.54)	41 (39.81)	61 (19.00)	
6–9 times	19 (3.18)	6 (3.47)	5 (4.85)	8 (2.49)	
≥10 times	3 (0.5)	0	2 (1.94)	1 (0.31)	
How often do you use online apps to meet new partners?					
Always	134 (22.45)	24 (13.87)	27 (26.21)	83 (25.86)	**0** **.** **026**
Occasionally	352 (58.96)	110 (63.58)	60 (58.25)	182 (56.7)	
Never	111 (18.59)	39 (22.54)	16 (15.53)	56 (17.45)	
Number of risky behaviours^b^					
0	3 (0.5)	0	1 (0.97)	2 (0.62)	0.858
1	122 (20.44)	38 (21.97)	18 (17.48)	66 (20.56)	
2	279 (46.73)	78 (45.09)	48 (46.6)	153 (47.66)	
3	193 (32.33)	57 (32.95)	36 (34.95)	100 (31.15)	

^a^ Chi-squared test (Fisher exact); Kruskal–Wallis test for non-parametric data.

^b^ defined based on the number of affirmative responses across three main domains: (1) sexual partners (having a partner they barely know or having a regular partner in an open relationship), (2) condom use (occasional or never), and (3) use of psychoactive substances during sex including chemsex practice (sometimes or always).

Values that differ significantly (*p* < 0.05) are marked in bold.

Most participants (71.0%) had heard of PrEP prior to this study, while 17.2% reported previous use. PrEP awareness differed significantly by testing setting (*p* = 0.011), with higher awareness in the NGO-based services. No significant differences in PrEP awareness were observed by age group or employment status, although participants aged 31–40 years were somewhat more represented among PrEP users (35.9%). There was a statistically significant difference in PrEP awareness across educational levels (*p* = 0.011)—previous PrEP users were most often university graduates (41.8%), whereas participants unaware of PrEP (41.6%) or who had heard but never used it (42.4%) predominantly had completed high school. Among participants who had not heard of PrEP, the largest share reported having no sexual partner (31.8%), whereas among those who had heard of PrEP, the majority—both users (30.1%) and non-users (29.6%)—reported a permanent sexual partner (*p* = 0.049).

Among PrEP users, 21.4% reported never using condoms compared with approximately 10% of non-users (*p* = 0.017). Occasional use of psychoactive substances during sex (including chemsex practice) was reported by 36.7% overall, with the highest prevalence among PrEP users (50.5%) compared with non-users (30.8%) and those unaware of PrEP (39.3%) (*p* = 0.003). Always using online apps was reported by 26.2% of PrEP users, compared with 13.9% of those unaware of PrEP and 25.9% of non-users.

In Table 2, 55.4% of participants demonstrated correct basic knowledge of PrEP, 28.8% incomplete basic knowledge, and 18.8% insufficient basic knowledge. Correct knowledge was significantly more common among unmarried participants (69.4%) and among previous PrEP users (30.2%) (*p* = 0.012 and *p* = 0.002, respectively). Overall, the majority of participants (80.2%) expressed a positive general attitude toward PrEP, while 11.7% were undecided and 8.1% reported a negative attitude. No significant differences in knowledge or attitude were observed by age or education, although the youngest group (18–25 years) tended to have slightly lower correct knowledge (23.4%), and university graduates were over-represented among those with correct knowledge (43.4%). Regarding risk behaviours, correct knowledge was associated with more frequent use of online apps to meet partners (28.1% vs. 17.9%; *p* = 0.027), and a positive attitude was associated with occasional use of psychoactive substances during sex (including chemsex practice) (49% vs. 4.1%; *p* = 0.024).

**Table 2:** Basic knowledge level and general attitude towards PrEP by selected characteristics of participants.

	Basic Knowledge level, *n* = 424				General attitude, *n* = 420^c^			
	Insufficient basic knowledge; *n* = 67 (18.80%)	Incomplete basic knowledge, *n* = 122 (28.77%)	Correct basic knowledge, *n* = 235 (55.42%)	*p*-value^a^	Negative attitude, *n* = 34 (8.10%)	Undecided, *n* = 49 (11.67)	Affirmative attitude, *n* = 337 (80.24)	*p*-value^a^
Testing setting								
Public health institute	23 (34.33)	30 (24.59)	55 (23.4)	0.188	10 (29.41)	6 (12.24)	92 (27.3)	0.069
NGO	44 (65.67)	92 (75.41)	180 (76.6)		24 (70.59)	43 (87.76)	245 (72.7)	
Age in years, mean (SD)	30.39 (8.80)	30.71 (8.13)	31.97 (8.08)	0.115	31.12 (7.98)	31.00 (8.16)	31.43 (8.26)	0.932
Age category								
18–25	27 (40.3)	39 (31.97)	55 (23.4)	0.148	7 (20.59)	15 (30.61)	97 (28.78)	0.586
26–30	11 (16.42)	29 (23.77)	66 (28.09)		12 (35.29)	13 (26.53)	81 (24.04)	
31–40	20 (29.85)	35 (28.69)	76 (32.34)		10 (29.41)	11 (22.45)	109 (32.34)	
>40	9 (13.43)	19 (15.57)	38 (16.17)		5 (14.71)	10 (20.41)	50 (14.84)	
The highest completed level of education								
Elementary school	2 (2.99)	3 (2.46)	5 (2.13)	0.283	2 (5.88)	1 (2.04)	7 (2.08)	0.642
High school	30 (44.78)	55 (45.08)	84 (35.74)		15 (44.12)	19 (38.78)	133 (39.47)	
College/Higher school	16 (23.88)	24 (19.67)	44 (18.72)		5 (14.71)	13 (26.53)	65 (19.29)	
Faculty	19 (28.36)	40 (32.79)	102 (43.4)		12 (35.29)	16 (32.65)	132 (39.17)	
Marital status								
Married	2 (2.99)	7 (5.74)	8 (3.4)	**0** **.** **012**	0	1 (2.04)	16 (4.75)	**0** **.** **047**
Unmarried	39 (58.21)	84 (68.85)	163 (69.36)		23 (67.65)	25 (51.02)	236 (70.03)	
Common-law partnership	22 (32.84)	23 (18.85)	36 (15.32)		6 (17.65)	17 (34.69)	57 (16.91)	
Divorced	2 (2.99)	5 (4.1)	25 (10.64)		5 (14.71)	5 (10.2)	21 (6.23)	
Widower	2 (2.99)	2 (1.64)	2 (0.85)		0	1 (2.04)	5 (1.48)	
Missing	0	1 (0.82)	1 (0.43)		0	0	2 (0.59)	
Working status								
Pupil/student	10 (14.93)	17 (13.93)	30 (12.77)	0.418	3 (8.82)	4 (8.16)	49 (14.54)	0.108
Employed	41 (61.19)	86 (70.49)	166 (70.64)		23 (67.65)	31 (63.27)	237 (70.33)	
Unemployed	16 (23.88)	18 (14.75)	34 (14.47)		8 (23.53)	13 (26.53)	46 (13.65)	
Missing	0	1 (0.82)	5 (2.13)		0	1 (2.04)	5 (1.48)	
*Risk assessment for STIs during the last year*								
During this period, I have:								
Permanent sexual partner	26 (38.81)	34 (27.87)	66 (28.09)	0.083	8 (23.53)	16 (32.65)	102 (30.27)	0.674
Permanent sexual partner with whom I am in an open relationship	18 (26.87)	24 (19.67)	69 (29.36)		10 (29.41)	14 (28.57)	86 (25.52)	
Sexual partners whom I barely know	9 (13.43)	34 (27.87)	59 (25.11)		10 (29.41)	7 (14.29)	84 (24.93)	
I don't have a sexual partner	14 (20.9)	30 (24.59)	41 (17.45)		6 (17.65)	12 (24.49)	65 (19.29)	
I use a condom during sexual intercourse								
Always	27 (40.3)	44 (36.07)	92 (39.15)	0.910	13 (38.24)	22 (44.9)	126 (37.39)	0.558
Occasionally	30 (44.78)	63 (51.64)	114 (48.51)		19 (55.88)	22 (44.9)	164 (48.66)	
Never	10 (14.93)	15 (12.3)	29 (12.34)		2 (5.88)	5 (10.2)	47 (13.95)	
During sexual intercourse, I use psychoactive substances (including chemsex practice)								
Always	1 (1.49)	1 (0.82)	2 (0.85)	0.868	0	2 (4.08)	2 (0.59)	**0** **.** **024**
Occasionally	21 (31.34)	44 (36.07)	86 (36.6)		14 (41.18)	24 (48.98)	111 (32.94)	
Never	45 (67.16)	77 (63.11)	147 (62.55)		20 (58.82)	23 (46.94)	224 (66.47)	
In the last year, how often have you been tested for HIV?								
Never	20 (29.85)	28 (22.95)	45 (19.15)	0.333	8 (23.53)	10 (20.41)	75 (22.26)	0.893
1–2 times	31 (46.27)	62 (50.82)	120 (51.06)		14 (41.18)	24 (48.98)	173 (51.34)	
3–5 times	12 (17.91)	28 (22.95)	62 (26.38)		11 (32.35)	13 (26.53)	77 (22.85)	
6–9 times	4 (5.97)	4 (3.28)	5 (2.13)		1 (2.94)	2 (4.08)	9 (2.67)	
≥10 times	0	0	3 (1.28)		0	0	3 (0.89)	
How often do you use online apps to meet new partners?								
Always	12 (17.91)	32 (26.23)	66 (28.09)	**0** **.** **027**	11 (32.35)	8 (16.33)	90 (26.71)	0.070
Occasionally	35 (52.24)	69 (56.56)	138 (58.72)		19 (55.88)	26 (53.06)	194 (57.57)	
Never	20 (29.85)	21 (17.21)	31 (13.19)		4 (11.76)	15 (30.61)	53 (15.73)	
Number of risky behaviours^b^								
0	1 (1.49)	1 (0.82)	1 (0.43)	0.783	0	2 (4.08)	1 (0.3)	
1	17 (25.37)	25 (20.49)	42 (17.87)		7 (20.59)	10 (20.41)	67 (19.88)	0.059
2	31 (46.27)	56 (45.9)	114 (48.51)		13 (38.24)	26 (53.06)	158 (46.88)	
3	18 (26.87)	40 (32.79)	78 (33.19)		14 (41.18)	11 (22.45)	111 (32.94)	
Have you ever used PrEP?								
No	60 (89.55)	97 (79.51)	164 (69.79)	**0** **.** **002**	24 (70.59)	35 (71.43)	259 (76.85)	0.545
Yes	7 (10.45)	25 (20.49)	71 (30.21)		10 (29.41)	14 (28.57)	78 (23.15)	

^a^ Chi-squared test (Fisher exact); Kruskal–Wallis test for non-parametric data.

^b^ Based on the number of affirmative responses across three main domains: (1) sexual partners (having a partner they barely know or having a regular partner in an open relationship), (2) condom use (occasional or never), and (3) use of psychoactive substances during sex including chemsex practice (sometimes or always);.

^c^ Missing response (*n* = 4); Values that differ significantly (*p* < 0.05) are marked in bold.

Additionally, among those with correct basic knowledge of PrEP, 28.1% reported always using online apps to meet new partners compared with 17.9% among those with insufficient knowledge (*p* = 0.027). Overall attitude toward PrEP was predominantly positive: 80.24% expressed a positive general attitude, 11.7% were undecided, and 8.1% expressed a negative attitude.

**Figure 2:**
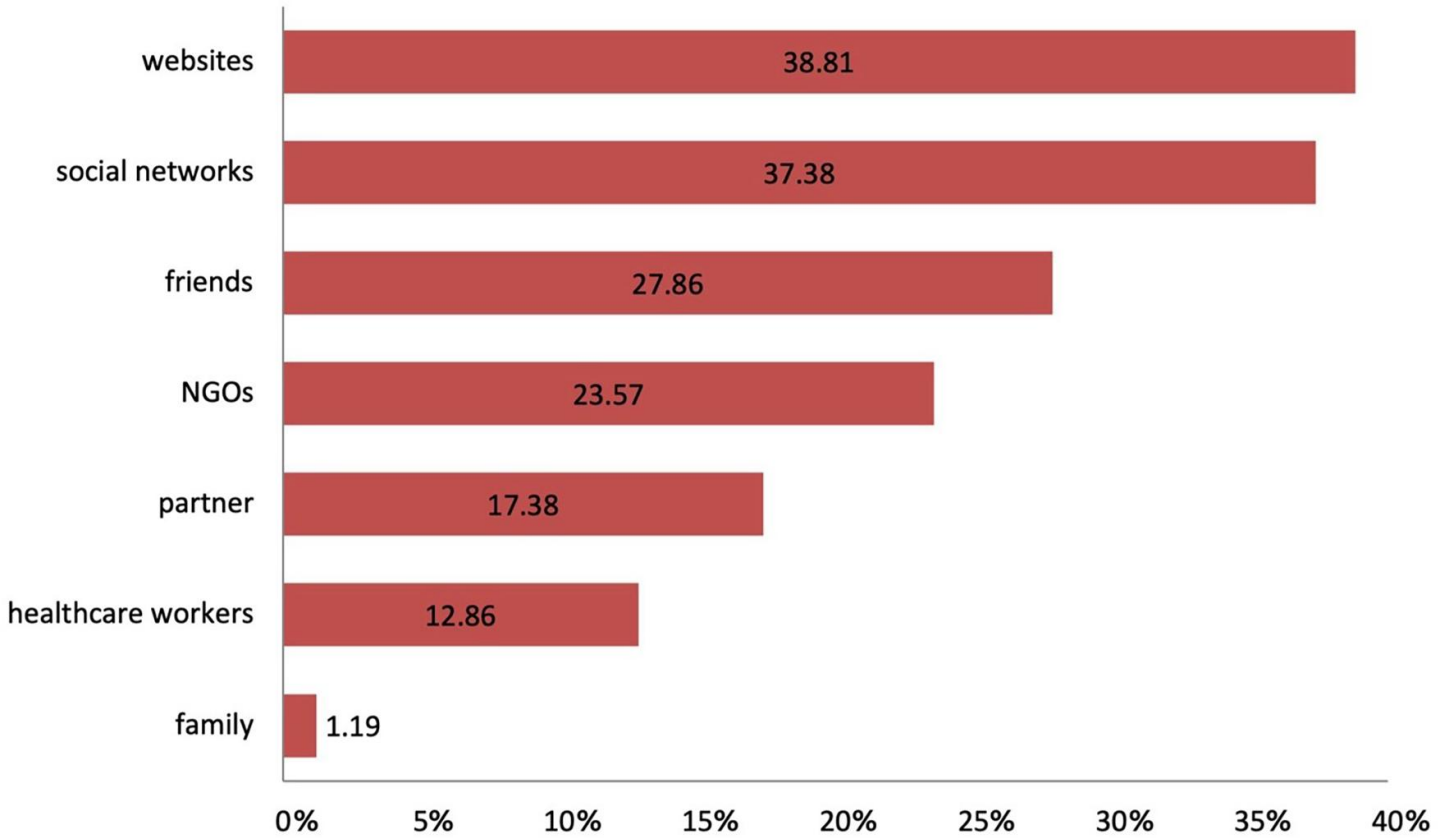
Sources of information on PrEP used by participants. Multiple answers were allowed.

Among participants who had heard of PrEP, the main information sources were websites (38.8%) and social network (37.4%) (Figure 2). Healthcare workers were cited least often, by about one in ten participants (12.9%).

**Figure 3:**
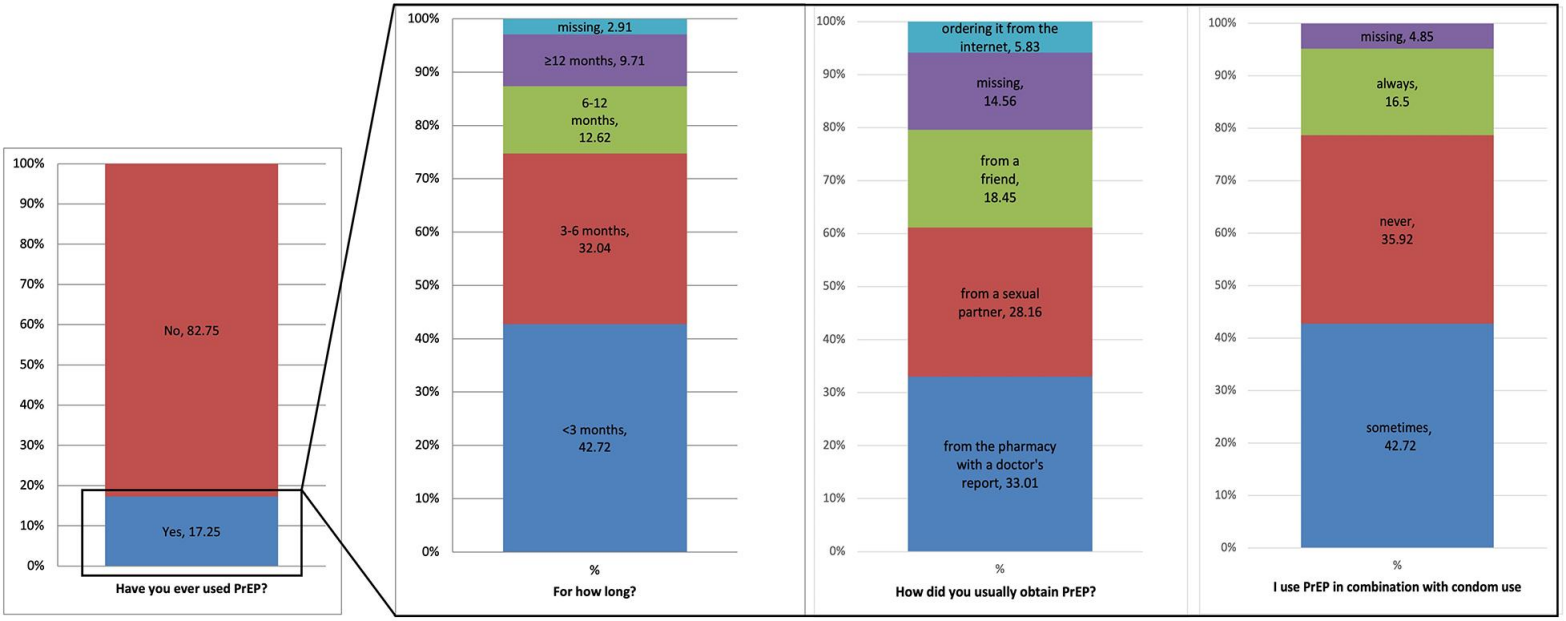
Experience with PrEP use among the surveyed participants.

Among those with PrEP use experience (*n* = 103; 17.2%), most reported using it for less than three months (*n* = 44; 42.7%) (Figure 3). The most common ways of acquiring PrEP were from a pharmacy with a medical prescription (33.0%) and from a sexual partner (28.2%). During PrEP use, 42.7% reported occasional condom use, while almost 36% reported never using condoms.

**Figure 4:**
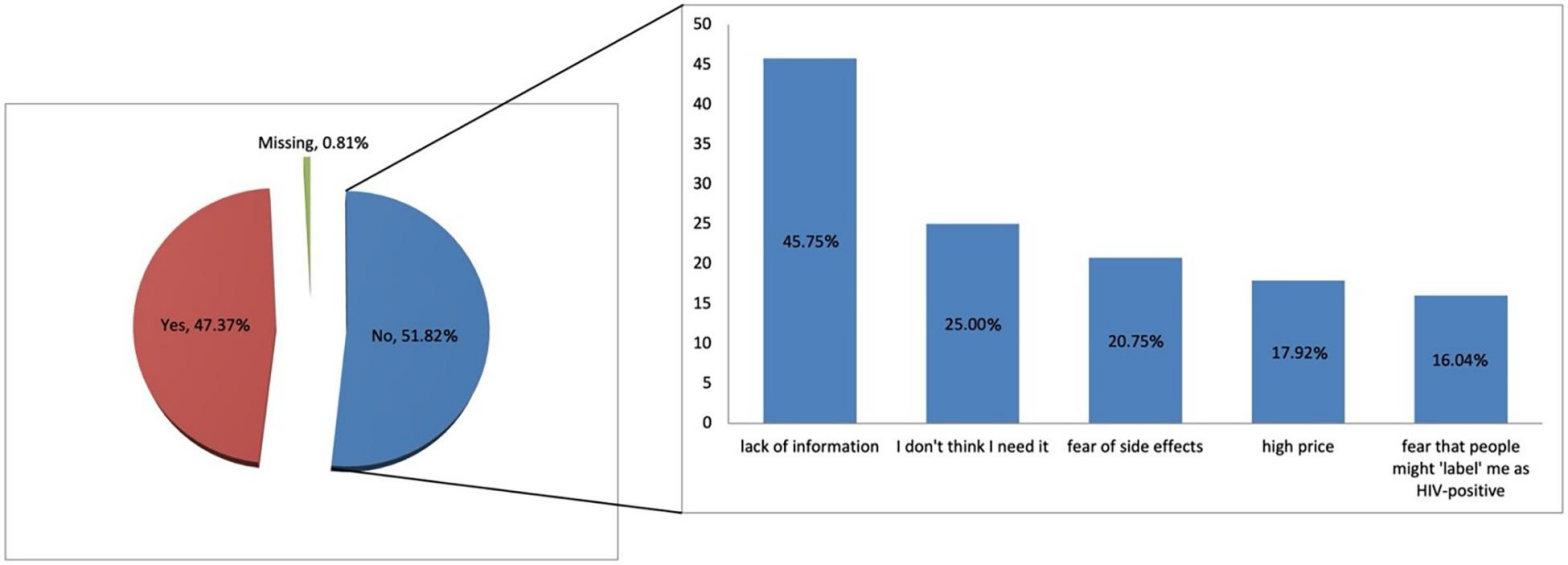
Barriers for not considering the use of PrEP as preventive measure against HIV.

Among those who had never used PrEP (*n* = 494; 82.7%), more than half had not previously considered using it for HIV prevention (*n* = 256; 51.8%). The main barriers were lack of information (*n* = 97; 45.8%), followed by low perceived need (“I don't think I need it”, *n* = 53; 25.0%) and fear of side effects (*n* = 44; 20.8%) (Figure 4).

**Figure 5:**
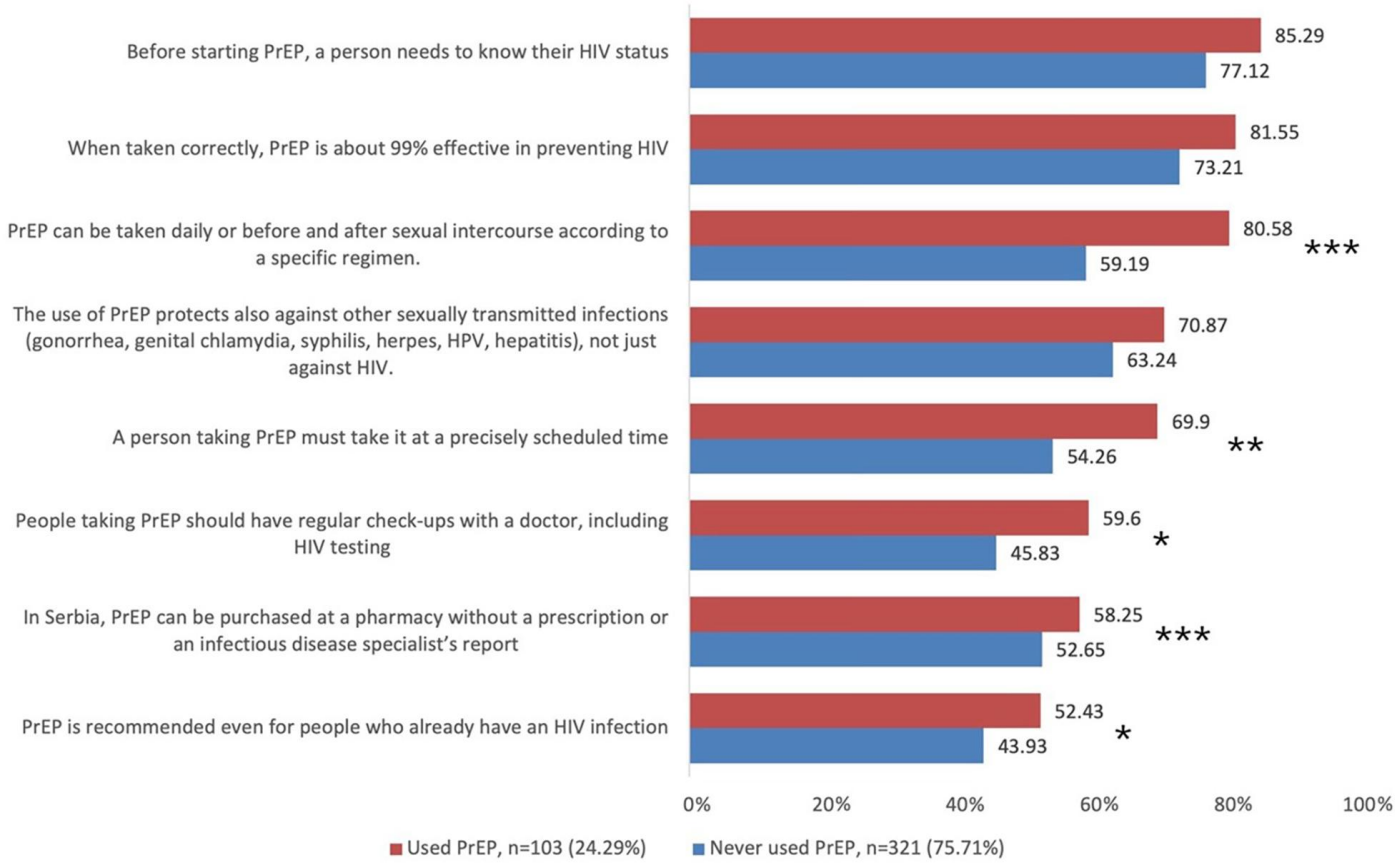
Share of correct answers in relation to the previous use of PrEP among participants that have heard of PrEP. Using Chi-square test; * *p* < 0.05; ** *p* < 0.01; *** *p* < 0.001.

Figure 5 presents the share of correct answers on PrEP-related items among PrEP previous users vs. never-users (limited to those who had heard of PrEP). Prior users consistently showed higher correct response proportions across all eight items. Significant differences were noted for: (i) need for medical monitoring including HIV testing every three months (59.6% vs. 45.8%, *p* = 0.018), (ii) taking PrEP regularly at a specific time (69.9% vs. 54.3%, *p* = 0.006), and (iii) the option to take PrEP daily or around sexual activity (80.6% vs. 59.2%, *p* < 0.001). The lowest correct response rates were observed for statements about (iv) availability of PrEP in Serbia without a prescription (58.3% vs. 52.7%, *p* < 0.001) and (v) recommending PrEP for people already living with HIV (52.4% vs. 43.9%, *p* = 0.016).

**Figure 6:**
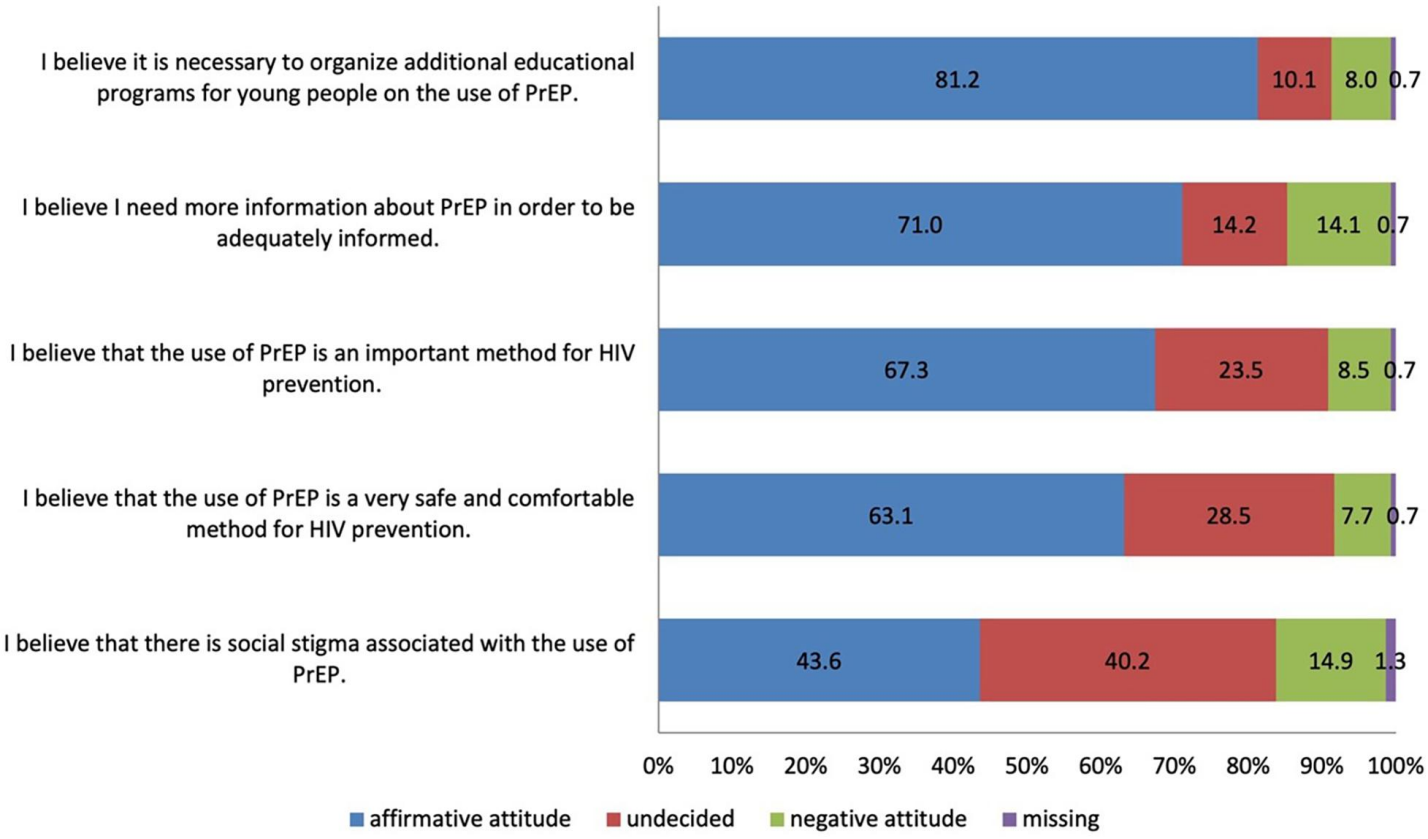
Prevalence of attitudes about the PrEP use among all surveyed participants.

Most participants expressed affirmative attitudes toward PrEP use, with around 67% stating that PrEP is important, as reported in the Figure 6. In addition, 81.2% believed that additional education on PrEP among young people is needed, and 71% reported the need for more information to feel adequately informed. Finally, 43.6% perceived a social stigma associated with the PrEP use.

When attitudes toward PrEP were compared across basic-knowledge levels, statistically significant differences were observed for most items, consistently favouring those with correct basic knowledge (Table 3). Around 87% of those with correct basic knowledge agreed that PrEP is an important method for HIV prevention, compared with 70.4% of those with incorrect basic knowledge (*p* < 0.001). Participants with incorrect knowledge were significantly more often undecided regarding this domain (20.0% vs. 4.7%). Similarly, 81.3% of those with correct knowledge viewed PrEP as a safe and comfortable method of HIV prevention compared with 66.1% among those with incorrect knowledge (*p* = 0.001).

**Table 3:** Prevalence of attitudes about the PrEP use according to the participants' basic knowledge about the PrEP.

	Basic knowledge^a^	Negative attitude	Undecided	Affirmative attitude	*Missing*	*p*-value
I believe that the use of PrEP is an important method for HIV prevention.	Incorrect	16 (8.47)	38 (20.11)	133 (70.37)	2 (1.06)	**<0.001**
	Correct	18 (7.66)	11 (4.68)	204 (86.81)	2 (0.85)	
I believe that the use of PrEP is a very safe and comfortable method for HIV prevention.	Incorrect	16 (8.47)	46 (24.34)	125 (66.14)	2 (1.06)	**0** **.** **001**
	Correct	13 (5.53)	29 (12.34)	191 (81.28)	2 (0.85)	
I believe that there is social stigma associated with the use of PrEP.	Incorrect	26 (13.76)	69 (36.51)	90 (47.62)	4 (2.12)	0.346
	Correct	39 (16.60)	71 (30.21)	121 (51.49)	4 (1.70)	
I believe I need more information about PrEP in order to be adequately informed.	Incorrect	17 (8.99)	21 (11.11)	149 (78.84)	2 (1.06)	**0** **.** **005**
	Correct	47 (20.00)	29 (12.34)	157 (66.81)	2 (0.85)	
I believe it is necessary to organize additional educational programs for young people on the use of PrEP.	Incorrect	14 (7.41)	23 (12.17)	150 (79.37)	2 (1.06)	**0** **.** **003**
	Correct	13 (5.53)	9 (3.83)	211 (89.79)	2 (0.85)	

^a^ Based on participants' answers on two domains: (a) When taken correctly, PrEP is about 99% effective in preventing HIV, (b) PrEP can be taken daily or before and after sex according to a specific scheme; participants were classified as having incorrect basic knowledge if reported affirmative answer on just one or when did not report affirmative answer to any of the two, while participants that reported affirmative answer on both were classified as having the correct basic knowledge. Values that differ significantly (*p* < 0.05) are marked in bold.

In models exploring predictors of correct basic knowledge (Table 4), older age categories had higher odds of correct knowledge (26–30 years: OR = 1.98, 95% CI: 1.16–3.37, *p* = 0.012; 31–40 years: OR = 1.66, 95% CI: 1.01–2.73, *p* = 0.047) relative to 18–25 years. Divorced participants had almost triple the odds (OR = 2.70, 95% CI: 1.13–6.43, *p* = 0.026), whereas those living in a common-law partnership had lower odds (OR = 0.60, 95% CI: 0.37–0.99, *p* = 0.046) compared with unmarried participants. Always using online apps to meet partners was associated with nearly double the odds of correct knowledge (OR = 1.98, 95% CI: 1.09–3.62, *p* = 0.026), similar to occasional app use (OR = 1.75, 95% CI: 1.03–2.99, *p* = 0.038), compared with those who never used apps. Previous PrEP use was also associated with higher odds of correct knowledge (OR = 2.12, 95% CI: 1.33–3.40, *p* = 0.002). Results remained substantially the same after adjusting for age and marital status.

**Table 4:** Factors associated with basic knowledge about the PrEP and with the affirmative attitude on the use of PrEP.

	Correct basic knowledge about the PrEP						Affirmative attitude on the use of PrEP					
	OR	95% CI	*p*-value	aOR	95% CI	*p*-value	OR	95% CI	*p*-value	aOR	95% CI	*p*-value
Age, years	1.02	0.99–1.05	0.089	-	-	-	1.01	0.98–1.04	0.704	-	-	-
Age category (years)												
18–25	ref.	-	-	-	ref.	-	-	-				
26–30	**1** **.** **98**	**1.16–3.37**	**0** **.** **012**	-	-	-	0.74	0.39–1.40	0.349	-	-	-
31–40	**1** **.** **66**	**1.01–2.73**	**0** **.** **047**	-	-	-	1.18	0.61–2.27	0.627	-	-	-
>40	1.63	0.89–2.98	0.114	-	-	-	0.76	0.36–1.58	0.459	-	-	-
The highest completed level of education												
Elementary school	Ref.			Ref.			Ref.			Ref.		
High school	0.99	0.28–3.54	0.985	0.93	0.26–3.37	0.914	1.68	0.41–6.83	0.471	1.33	0.32–5.61	0.693
College/Higher school	1.10	0.30–4.08	0.887	1.02	0.27–3.85	0.976	1.55	0.36–6.60	0.555	1.29	0.29–5.72	0.734
Faculty	1.73	0.48–6.22	0.402	1.53	0.42–5.60	0.523	2.02	0.49–8.30	0.329	1.67	0.39–7.16	0.492
Marital status												
Married	0.67	0.25–1.79	0.425	-	-	-	3.25	0.42–25.13	0.258	-	-	-
Unmarried	Ref.			-	-	-	Ref.			-	-	-
Common-law partnership	**0** **.** **6**	**0.37–0.99**	**0** **.** **046**	-	-	-	**0** **.** **50**	**0.28–0.90**	**0** **.** **020**	-	-	-
Divorced	**2** **.** **7**	**1.13–6.43**	**0** **.** **026**	-	-	-	**0** **.** **43**	**0.19–0.96**	**0** **.** **041**	-	-	-
Widower	0.38	0.07–2.09	0.265	-	-	-	1.02	0.12–8.90	0.988	-	-	-
Working status												
Pupil/student	0.86	0.48–1.50	0.576	1.05	0.55–1.99	0.879	1.59	0.68–3.71	0.279	1.58	0.63–3.98	0.334
Employed	Ref.			Ref.			Ref.			Ref.		
Unemployed	0.77	0.45–1.30	0.321	0.84	0.48–1.47	0.550	**0** **.** **50**	**0.28–0.90**	**0** **.** **022**	0.58	0.31–1.10	0.093
*Risk assessment for STIs during the last year*												
During this period, I have:												
Permanent sexual partner	Ref.			Ref.			Ref.			Ref.		
Permanent sexual partner with whom I am in an open relationship	1.49	0.89–2.51	0.130	1.46	0.86–2.48	0.164	0.84	0.45–1.59	0.598	0.99	0.52–1.91	0.985
Sexual partners whom I barely know	1.25	0.74–2.11	0.410	1.25	0.74–2.13	0.401	1.16	0.59–2.31	0.666	1.12	0.56–2.24	0.755
I don't have a sexual partner	0.85	0.49–1.47	0.555	0.9	0.51–1.58	0.715	0.85	0.43–1.69	0.641	0.84	0.41–1.70	0.620
I use a condom during sexual intercourse												
Always	1.12	0.60–2.07	0.726	1.14	0.61–2.15	0.684	0.54	0.22–1.29	0.164	0.47	0.19–1.15	0.099
Occasionally	1.06	0.58–1.93	0.857	1.05	0.57–1.94	0.880	0.60	0.25–1.41	0.240	0.51	0.21–1.24	0.138
Never	Ref.			Ref.			Ref.			Ref.		
During sexual intercourse, I use psychoactive substances (including chemsex practice)												
Always	0.83	0.12–5.98	0.853	0.77	0.11–5.61	0.796	0.19	0.03–1.40	0.104	0.22	0.03–1.79	0.157
Occasionally	1.1	0.73–1.64	0.648	1.09	0.71–1.67	0.686	**0** **.** **56**	**0.34–0.92**	**0** **.** **021**	0.67	0.40–1.13	0.137
Never	Ref.			Ref.			Ref.			Ref.		
In the last year, how often have you been tested for HIV?												
Never	Ref.			Ref.			Ref.			Ref.		
1–2 times	1.38	0.84–2.24	0.200	1.35	0.83–2.22	0.227	1.09	0.59–2.04	0.780	1.11	0.59–2.08	0.753
3–5 times	1.66	0.94–2.92	0.083	1.64	0.92–2.92	0.096	0.77	0.39–1.53	0.457	0.90	0.45–1.83	0.780
>5 times	1.07	0.37–3.08	0.905	1.08	0.37–3.15	0.894	0.96	0.24–3.76	0.953	1.23	0.31–4.97	0.769
How often do you use online apps to meet new partners?												
Always	**1** **.** **98**	**1.09–3.62**	**0** **.** **026**	**1.97**	**1.07–3.61**	**0** **.** **029**	1.70	0.83–3.49	0.150	1.55	0.75–3.22	0.238
Occasionally	**1** **.** **75**	**1.03–2.99**	**0** **.** **038**	**1.77**	**1.04–3.02**	**0** **.** **036**	1.55	0.83–2.86	0.166	1.55	0.83–2.90	0.166
Never	Ref.			Ref.			Ref.			Ref.		
Number of risky behaviours^a^												
0	Ref.			Ref.			Ref.			Ref.		
1	2.00	0.17–22.91	0.577	2.12	0.18–24.78	0.549	7.88	0.67–92.15	0.100	5.29	0.41–68.37	0.202
2	2.62	0.23–29.37	0.435	2.74	0.24–31.22	0.418	8.1	0.72–91.66	0.091	5.58	0.45–69.58	0.182
3	2.69	0.24–30.38	0.424	2.67	0.23–30.58	0.429	8.88	0.77–101.81	0.079	6.56	0.52–82.57	0.146
Have you ever used PrEP?												
No	Ref			Ref.			Ref.			Ref.		
Yes	**2.12**	**1.33–3.40**	**0.002**	**2.08**	**1.29–3.36**	**0.003**	0.74	0.43–1.27	0.273	0.80	0.46–1.38	0.416
Basic knowledge level												
Insufficient basic knowledge	NA	NA	NA	NA	NA	NA	ref.	ref.				
Incomplete basic knowledge	NA	NA	NA	NA	NA	NA	1.20	0.63–2.31	0.578	1.08	0.56–2.11	0.818
Correct basic knowledge	NA	NA	NA	NA	NA	NA	**3** **.** **21**	**1.68–6.12**	**<0** **.** **001**	**3** **.** **08**	**1.60–5.94**	**0** **.** **001**

^a^ Based on the number of affirmative responses across three main domains: (1) sexual partners (having a partner they barely know or having a regular partner in an open relationship), (2) condom use (occasional or never), and (3) use of psychoactive substances during sex including chemsex practice (sometimes or always).

Values that differ significantly (*p* < 0.05) are marked in bold.

aOR, adjusted OR for age and marital status; ref., reference category.

On the other hand, in the models examining predictors of an affirmative attitude toward PrEP, correct basic knowledge was the strongest predictor (OR = 3.21, 95% CI: 1.68–6.12, *p* < 0.001). Participants who occasionally use psychoactive substances during sex (including chemsex practice) had lower odds of an affirmative attitude (OR = 0.56, 95% CI: 0.34–0.92, *p* = 0.021) compared with those who never practiced it.

## 4 Discussion

To the best of our knowledge, this cross-sectional study is the first study to assess knowledge and attitudes related to PrEP in Serbia, and one of the largest PrEP-related surveys conducted in the Central and Eastern European region. This study included 28.3% (*n* = 597) of all counselled and/or tested clients across nine recruiting centres in the AP Vojvodina. The mean age of participants was 31.7 years (SD = 8.64), with the majority having completed high school as their highest level of education (40.4%). Approximately 70% of respondents had heard of PrEP prior to this research, yet only 17.2% reported previous use. Notably, PrEP use was more common among university-educated participants (41.8%), whereas those with only secondary education predominated among both non-users and individuals unaware of PrEP (42.4% and 41.6%, respectively).

The proportion of respondents who had heard of PrEP in this study (around 70%) is higher than that reported in several other European settings, yet actual use remains comparably low (16, 20). For instance, in Portugal, only 10.9% of key populations were aware of PrEP and less than 1% had ever used it (20), while in Italy, 87% of MSM were familiar with PrEP but only 7.5% reported use (16). Additionally, in comparison with the PrEP cascade reported by Wirawan et al. (19) among adult MSM who self-identified as HIV-negative and were resident in Asia, the PrEP awareness in our study was lower (71% vs. 82%), as it was the lifetime PrEP use among PrEP-aware participants (24.3% vs. 35%), indicating a larger awareness-to-uptake gap in our setting. Engagement in behaviors associated with an increased risk of HIV acquisition among PrEP-naïve participants was substantially higher in our sample (99.4%) than reported by Wirawan et al. (55%). Despite this near-universal risk exposure, consideration of PrEP in our study (63.3%) remained lower than willingness to use PrEP observed by Wirawan et al. (74%), suggesting that barriers such as structural, access- or provider-related factors, may have limited PrEP consideration and the PrEP uptake. Similar discrepancies between awareness and uptake have also been observed in China, where just 22.7% of MSM had heard of PrEP, although almost 90% expressed willingness to use it if available (15). Such differences suggest that awareness alone does not translate into practice and that behavioural, structural, and systemic barriers remain pervasive across diverse contexts. Despite increasing awareness, the low level of PrEP uptake observed in this study mirrors patterns in CEE, where implementation has been hindered by multiple structural, social, and cultural barriers (4, 5, 8). Limited availability of PrEP through national health systems, insufficient engagement of healthcare professionals, and inconsistent inclusion of PrEP in HIV-prevention strategies continue to restrict its broader adoption. Furthermore, sustainable scale-up of PrEP requires not only political commitment but also public financing and integration into existing healthcare systems. Evidence from the United Kingdom has shown that PrEP is a cost-effective intervention among MSM, providing substantial long-term savings by reducing new HIV infections (7).

In Serbia, PrEP uptake challenges are compounded by stigma and moralizing public discourse surrounding sexuality and HIV, which discourage individuals, particularly MSM, from seeking preventive services (11, 12). Similarly, studies from Western Europe emphasize that even when PrEP is accessible, social stigma, misconceptions about side effects, and low self-perceived HIV risk remain key deterrents (16, 21). These barriers collectively underscore the need for comprehensive and inclusive prevention strategies that extend beyond biomedical availability to address social determinants of health and community-level acceptance. Importantly, these structural and cultural barriers shape individual risk perception and behavioural choices, which often manifest as reduced engagement with formal HIV prevention services, including delayed or infrequent HIV testing, reliance on informal information sources, and inconsistent use of preventive counselling (4, 16, 20). In this study, nearly all participants (99.5%) reported at least one risky sexual behaviour during the last year, indicating that the surveyed population represents a group with substantial exposure to HIV and other STIs. Among PrEP users, 21.4% reported never using condoms, while occasional use of psychoactive substances during sex (including chemsex practice) was reported by one-third of participants, with the highest proportion among those who had used PrEP (50.5%). Every fourth respondent had not been tested for HIV within the past year, and approximately half reported occasional use of online applications to meet sexual partners. These findings are consistent with behavioural patterns reported among key populations in other European countries, where PrEP users tend to engage in higher-risk sexual practices, often combining PrEP with inconsistent condom use and psychoactive substance use during sex (5, 8). Importantly, evidence from high HIV-burden settings further demonstrates that gaps in PrEP knowledge, attitudes, and practices are not confined to low-prevalence regions. A recent systematic review including studies from South Africa, Ethiopia, and other high-incidence countries showed that inadequate PrEP knowledge and ambivalent or negative attitudes persist even among healthcare providers, and that limited knowledge consistently predicts lower acceptability and weaker implementation of PrEP services (6). Qualitative evidence from West Africa complements these findings, showing that despite high awareness, stigma, misinformation, concerns about side effects, and anticipated discrimination substantially limit PrEP uptake among GBMSM in Ghana (22). Although PrEP is a critical biomedical prevention tool, these results highlight the need to integrate PrEP delivery with broader sexual-health education, adherence and risk-reduction counselling (23, 24).

More than half of the participants (55.4%) demonstrated a satisfactory level of basic knowledge about PrEP, while 28.8% had incomplete and 18.8% insufficient knowledge. Among those with incorrect knowledge, only about 10% had previously used PrEP, compared with 30% among participants with satisfactory knowledge, suggesting that prior use contributes to a better understanding of its function and effectiveness. Similar findings have been reported in other European studies, where individuals with prior exposure to PrEP or higher health literacy are more likely to possess accurate knowledge of its preventive role (4, 6). Nonetheless, persistent misconceptions remain, particularly regarding eligibility and medical follow-up requirements (8, 18). This underscores the importance of targeted, provider-led education to strengthen accurate understanding of PrEP among both potential users and healthcare workers. The vast majority of participants (80.2%) expressed a positive general attitude toward PrEP, while 11.7% were undecided and only 8.1% reported a negative attitude. Such widespread affirmative perception indicates growing openness to biomedical prevention within key populations, though not necessarily translating into uptake. Similar findings have been reported in other European contexts, where positive attitudes coexist with limited use, largely due to systemic barriers, stigma, and constrained service access (5, 8, 20). These results suggest that favourable attitudes can provide a foundation for future scale-up, provided that knowledge gaps and healthcare engagement are addressed through national HIV-prevention strategies.

Participants who had previously used PrEP demonstrated higher proportions of correct answers across all knowledge domains compared to non-users, confirming that direct experience with PrEP contributes to improved understanding of its use and monitoring. Nevertheless, notable misconceptions persisted, particularly regarding its availability in Serbia, where 58.3% of respondents incorrectly believed PrEP could be obtained without medical prescription, and incorrectly believed that it is also intended for people living with HIV. Comparable gaps have been observed in the UK and China, where users displayed better overall knowledge but ongoing uncertainty about medical supervision and eligibility (15, 21). These findings indicate that experience alone is not sufficient to ensure correct knowledge and highlight the importance of structured education and physician-led counselling as integral components of PrEP delivery (6, 24). Among participants who had previously used PrEP, most reported using it for less than three months, typically obtaining it either from pharmacies with a medical prescription or from a sexual partner. Condom use during PrEP use was inconsistent—while many used it occasionally, more than one-third (35.9%) reported never using condoms when on PrEP. The internet and social media were the primary sources of information about PrEP (38.8% and 37.4%, respectively), whereas only one in ten participants cited healthcare workers as their main source. This gap mirrors evidence that community and online sources often substitute for formal provider communication, consistent with findings from other countries where online platforms, peers, and community networks often replace formal healthcare guidance (4, 11, 21). Among non-users, lack of information (45.8%), low perceived need, and fear of side-effects were the main barriers—findings consistent with international studies identifying limited knowledge, stigma, and healthcare distrust as major obstacles even in settings where PrEP is widely available (20). Strengthening communication between healthcare providers and NGOs and at-risk populations, alongside improving PrEP literacy through credible online campaigns, could therefore represent an essential step in increasing both awareness and adherence.

Most participants expressed affirmative attitudes, with over two-thirds agreeing that PrEP is important for HIV prevention. Furthermore, 81% believed that additional education should be organized, and 71% reported needing more information to feel adequately informed and able to make decisions regarding its use. However, nearly half perceived social stigma associated with PrEP use, suggesting that psychosocial and cultural barriers may continue to hinder open discussion and uptake (5, 6, 8). Studies among MSM in the United Kingdom and Central Europe indicate that concerns about being judged, labelled promiscuous, or disclosing sexual orientation continue to limit PrEP uptake (11, 21). Targeted educational initiatives, stigma reduction campaigns, and engagement of healthcare professionals as trusted sources of information are therefore critical to translating positive attitudes into actual preventive behaviour. A statistically significant association was observed between paticipants' knowledge level and attitudes: those with correct basic knowledge were far more likely to express positive attitudes (87%) compared to those with incorrect knowledge (70.4%). This confirms that accurate understanding is a key determinant of acceptance (15, 16, 21, 22, 25). Comparable evidence from studies among MSM in Europe and Asia shows that awareness of PrEP's efficacy and dosing regimens predicts both acceptability and intended use (5, 26, 27). These findings underline the importance of comprehensive PrEP education strategies, not only to increase factual understanding but also to challenge stigma and hesitation associated with its use.

Older age groups demonstrated significantly higher odds of possessing correct basic knowledge about PrEP compared with the youngest participants. Divorced individuals were nearly three times more likely to have better knowledge, while those in common-law partnerships had lower odds compared with unmarried respondents. Furthermore, the use of online applications for meeting partners emerged as a positive predictor of accurate PrEP knowledge, likely reflecting greater exposure to sexual health information within digitally active networks. In contrast, the strongest predictor of a positive attitude toward PrEP was correct basic knowledge itself, emphasizing the crucial role of education in shaping favourable perceptions. Occasional engagement in use of psychoactive substances during sex (including chemsex practice), however, was negatively associated with affirmative attitudes, suggesting that individuals involved in higher-risk behaviours may face psychological or contextual barriers that undermine trust or interest in biomedical prevention. Similar associations between demographic, behavioural, and psychosocial determinants of PrEP awareness and acceptance have been reported in other European and global studies (5, 20, 28–30). These findings suggest that digital platforms and community-based, peer-networks could serve as valuable tools for disseminating accurate PrEP information, particularly among younger and more digitally engaged populations. Given that the use of online applications correlated with higher PrEP knowledge, targeted awareness campaigns through dating apps and social media may represent an effective strategy for reaching key populations. Simultaneously, collaboration with non-governmental organizations, which already play a major role in counselling and testing, can help bridge gaps between awareness and actual uptake.

Healthcare professionals should be actively involved in educational efforts, as their current role as information sources remains limited. Structured training programs for clinicians and public health practitioners could strengthen their capacity to communicate about PrEP confidently and without stigma. Importantly, within the Serbian healthcare system, clinicians play a central role in legitimizing PrEP as a credible and acceptable prevention option, thereby directly influencing PrEP literacy, trust, and subsequent uptake. While community-based organizations remain essential for outreach and demand generation, clinicians are uniquely positioned to translate awareness into uptake through clinical endorsement and prescribing authority. Taken together, such measures may enhance PrEP literacy, reduce misconceptions, and promote more equitable access across different subpopulations in AP Vojvodina and the wider CEE. Evidence from CEE, as well as from global contexts, similarly indicates that limited preparedness among healthcare professionals and insufficient integration of PrEP-related content into medical and pharmacy curricula remain major barriers to implementation (17, 18, 24, 31, 32). Strengthening professional training and awareness among physicians and pharmacists is particularly relevant in Serbia, where prescribing authority is restricted to infectious disease specialists, while pharmacists serve an important advisory, but not prescriptive, role (12, 17).

### 4.1 Implications for practice

Our results point to multiple actionable priorities for improving PrEP uptake and literacy in Serbia and the broader CEE region. First, integration of PrEP into national HIV-prevention frameworks is crucial, accompanied by coherent clinical guidelines, public financing, and structured referral pathways between primary healthcare and the specialized physicians authorized to prescribe PrEP (infectologist, epidemiologists, and related specialists). Strengthening the health system through clinician training, standardized protocols, and inclusion of PrEP in electronic medical record systems has been shown to increase prescribing confidence and reduce stigma in other European contexts (17, 24, 32).

Second, community and digital outreach should be strategically employed. Given that online applications and social media emerged as major sources of PrEP information in this study, digital campaigns and collaborations with dating apps could efficiently reach younger and higher-risk populations. Similar approaches have demonstrated measurable gains in awareness and willingness in studies from Europe, China, and Ghana (15, 22, 28). NGOs already play a central role in counselling, testing, and PrEP promotion; therefore, continued partnership and funding stability are essential to sustain these entry points (4, 8).

While community organizations remain essential for outreach and demand generation, clinicians are uniquely positioned to translate awareness into uptake through clinical endorsement and prescribing authority. This distinction is now more clearly articulated, particularly in the Serbian context, where prescribing authority is restricted to infectious disease specialists.

Third, stigma reduction and public education should be prioritized through communication campaigns addressing moral and identity-related misconceptions. Research in Serbia and the UK shows that perceived stigma, association with promiscuity, and fear of social judgment remain key obstacles (12, 21). Establishing PrEP as a responsible preventive practice—alongside condoms, regular testing, and harm reduction—can help expand acceptance across both key populations and general audiences.

Finally, capacity building for pharmacists and other healthcare professionals is important even though pharmacists in Serbia cannot prescribe PrEP. By improving their knowledge and communication skills, they can serve as reliable intermediaries who guide clients toward specialist care. Combined, these interventions could enhance PrEP literacy, reduce stigma, and improve equitable access to prevention services across diverse population groups.

### 4.2 Limitations and generalizability

This study has several limitations that should be considered when interpreting its findings. First, its cross-sectional design limits causal inference between knowledge, attitudes, and behavioural determinants of PrEP use. Second, the study relied on self-reported data, which may be subject to recall bias and social desirability bias, especially given the sensitivity of sexual behaviour and HIV-related topics. Additionally, given the sensitivity and potential stigma surrounding sexual orientation and same-sex behaviours in the social and cultural context, these data were not collected. Participants may have been reluctant to disclose this information which could have affected willingness to participate or led to socially desirable responses. Nevertheless, evidence from similar community-based HIV/STI testing settings suggests that MSM often represent a substantial proportion of individuals seeking testing and related services, therefore, our findings may be comparable with those reported in other studies. Third, while a combination of quantitative and qualitative approach could have provided additional insight into the contextual and explanatory aspects underlying the evidence, this was not feasible within the scope of the current study due to time and resource constraints that prevented the collection of primary qualitative data. Future research using qualitative methods such as interviews or focus groups or the mixed-methods approaches is recommended to complement, extend, and further deepen understanding of the findings. Fourth, the sampling frame was restricted to individuals attending voluntary counselling and testing centres or NGO-based community checkpoints, which may overrepresent persons already engaged with HIV prevention services and underestimate levels of ignorance or stigma in the general population. Finally, as demographic data were collected only from individuals who consented to participate in the study, a comparison with all counselled/tested individuals could not be performed, limiting our ability to assess potential selection bias.

Nevertheless, the inclusion of a large, regionally diverse sample across nine centres and collaboration between public and community sectors strengthen the validity and representativeness of the findings within the context of AP Vojvodina. Similar methodological designs have been used in studies assessing PrEP knowledge and attitudes in Portugal, Italy, and China (15, 16, 20), and the observed patterns align closely with trends reported across Central and Eastern Europe (4, 5, 8), supporting the generalisability of the results to comparable regional settings.

## 5 Conclusions

This study provides the first regional insight into knowledge and attitudes toward PrEP among sexually active male population in AP Vojvodina and Serbia. While awareness was relatively high, actual use remained limited, reflecting persistent gaps in access, provider engagement, and public understanding. Knowledge accuracy was strongly associated with positive attitudes, highlighting the transformative role of education and counselling in fostering PrEP acceptance. At the same time, stigma, low perceived need, and misinformation continue to impede uptake even among high-risk groups. To translate awareness into action, PrEP must be positioned as part of an integrated HIV-prevention strategy, linking healthcare providers, community networks, and digital platforms. Strengthening public health capacity, reducing stigma, and improving information flow between the healthcare system and at-risk populations represent essential steps toward more effective, equitable, and evidence-based HIV prevention in Serbia and the wider CEE region.

## Data Availability

The raw data supporting the conclusions of this article will be made available by the authors, without undue reservation.
